# Experienced migratory songbirds do not display goal-ward orientation after release following a cross-continental displacement: an automated telemetry study

**DOI:** 10.1038/srep37326

**Published:** 2016-11-23

**Authors:** Dmitry Kishkinev, Dominik Heyers, Bradley K. Woodworth, Greg W. Mitchell, Keith A. Hobson, D. Ryan Norris

**Affiliations:** 1 Department of Integrative Biology, University of Guelph, 50 Stone Road East, N1G 2W1 Guelph, Ontario, Canada; 2 School of Biological Sciences, Bangor University, Deiniol Road, LL57 2UW Bangor, Gwynedd, UK; 3 AG Neurosensorik / Animal Navigation, Institute of Biological and Environmental Sciences, University Oldenburg, D-26111 Oldenburg, Germany; 4 Wildlife Research Division, Environment and Climate Change Canada, National Wildlife Research Centre, 1125 Colonel By Drive, K1H 0H3, Canada, Ottawa, Ontario, Canada; 5 Wildlife Research Division, Environment and Climate Change Canada, 11 Innovation Boulevard, S7N 3H5 Saskatoon, Saskatchewan, Canada; 6 Department of Biology, University of Western Ontario, N6A 5B7, London, Ontario, Canada

## Abstract

The ability to navigate implies that animals have the capability to compensate for geographical displacement and return to their initial goal or target. Although some species are capable of adjusting their direction after displacement, the environmental cues used to achieve this remain elusive. Two possible cues are geomagnetic parameters (*magnetic map hypothesis*) or atmospheric odour-forming gradients (*olfactory map hypothesis*). In this study, we examined both of these hypotheses by surgically deactivating either the magnetic or olfactory sensory systems in experienced white-throated sparrows (*Zonotrichia albicollis*) captured in southern Ontario, Canada, during spring migration. Treated, sham-treated, and intact birds were then displaced 2,200 km west to Saskatchewan, Canada. Tracking their initial post-displacement migration using an array of automated VHF receiving towers, we found no evidence in any of the groups for compensatory directional response towards their expected breeding grounds. Our results suggest that white-throated sparrows may fall back to a simple constant-vector orientation strategy instead of performing true navigation after they have been geographically displaced to an unfamiliar area during spring migration. Such a basic strategy may be more common than currently thought in experienced migratory birds and its occurrence could be determined by habitat preferences or range size.

The mechanisms that control how millions of migratory birds successfully navigate over thousands of kilometers each year has fascinated biologists for centuries^1^. True navigation is the ability to find correct direction leading to the target destination even from unfamiliar sites. This may be achieved by at least two methods. One way is for animals to detect their position on the globe using a bi-coordinate (or multi-coordinate) detection system in which individuals can detect variation in both latitude and longitude^2^,^3^,^4^. Alternatively, navigation can be achieved by an individual assessing its position relative to the goal or target without specific knowledge of geographical coordinates^5^,^6^. In either case, animals capable of navigating should have the ability to compensate for geographical displacement and return to their target. Although several experiments have provided evidence that experienced birds (i.e. those that have finished at least one migration) are able to compensate for long-distance translocation^7^,^8^,^9^,^10^,^11^, how these birds can sense changes in position is unclear. The ‘map-and-compass’ concept^12^,^13^ suggests a two-step process: first, an animal detects its current position relative to the goal using a ‘map sense’ and then maintains a chosen direction to a goal using a ‘compass sense’. The nature of a ‘compass sense’ can be based on solar cues^13^,^14^, stellar cues^15^,^16^, or the geomagnetic field^17^, but the nature of a ‘map sense’ used to obtain positional information largely remains a mystery, at least for migratory landbirds.

Currently, there are two non-mutually exclusive hypotheses to explain how birds detect position. The *magnetic navigation hypothesis* proposes that animals use the Earth’s magnetic field parameters because of their relatively predictable spatial distribution^18^,^19^. In many regions of the world, the intensity of the geomagnetic field and inclination (the angle between the magnetic line and horizon) generally varies along a north-south axis whereas declination (the angle between magnetic and geographic poles) varies primarily along an east-west axis (http://www.ngdc.noaa.gov/geomag). Recent displacement studies provide support for this hypothesis in songbirds^20^,^21^. Magnetoreceptors associated with the ophthalmic branch of the trigeminal nerve have been suggested as likely candidates allowing birds to sense positional information^22^,^23^,^24^, although this has been debated^25^.

In contrast, the *olfactory navigation hypothesis* proposes that birds make use of odours or any volatile compounds in the atmosphere (perceived through olfactory receptors and the olfactory nerve), which form gradients stable enough to provide navigational performance^26^. Support for this hypothesis comes, in part, from displacement experiments on homing pigeons (*Colubma livia domestica*) and shearwaters (Procellariiformes) where olfactory deprivation led to reduced navigational ability^27^,^28^,^29^,^30^. A recent displacement study on lesser black-backed gulls (*Larus fuscus*) suggested that experimentally-induced anosmia led to impaired navigation performance in one of two release sites^10^. For migratory landbirds, experimental tests of the navigational role of olfactory sense have only been done in three species, two of which led to clear impairment of navigation performance (common swifts, *Apus apus*^31^, European starlings, *Sturnus vulgaris*^32^). Results of the third on gray catbirds (*Dumetella carolinensis*) were ambiguous because of high variability in flight directions in a relatively small sample size^33^. Interestingly, the latter study is the only one to date where both magnetic and olfactory mechanisms have been simultaneously tested in a songbird. Thus, more studies that examine both hypotheses are required before general conclusions can be made about the sensory mechanisms of bird navigation.

Here, we examined both the magnetic and olfactory navigation hypotheses by tracking spring migratory directions of experienced (at least one return migratory journey) white-throated sparrows (*Zonotrichia albicollis,* hereafter WTSPs) that were displaced 2,200 km from southern Ontario to Saskatchewan, Canada. We used three independent methods to determine the expected migratory directions of WTSPs captured in southern Ontario. Using another group of WTSPs captured at the same time, we either surgically deactivated the trigeminal (putative magnetic map) or olfactory (putative olfactory map) nerves. We also performed two ‘sham’ surgeries associated with each treatment and included a fifth group of intact (non-surgically-treated) birds. All WTSPs were then displaced to central Saskatchewan and tracked using an array of automated VHF towers to measure their migratory direction.

Following the *magnetic navigation hypothesis*, we predicted that birds without a functional V1 nerve would not adjust for the displacement, whereas all of the other groups with intact V1 nerves would shift their orientation towards the inferred migratory goal. Similarly, following the *olfactory navigation hypothesis*, we predicted that birds without a functional sense of smell would not compensate, whereas all of the other groups would adjust to the displacement by shifting their orientation. Finally, if all treated and non-treated birds flew north as expected from their Ontario capture site, this would suggest that WTSPs with migratory experience do not perform true navigation during spring migration.

## Material and Methods

### Ethical statement

All experiments were approved and conducted according to relevant legislation and guidelines (see Acknowledgements for the list of permits).

### Study species and trapping

**Figure 1:**
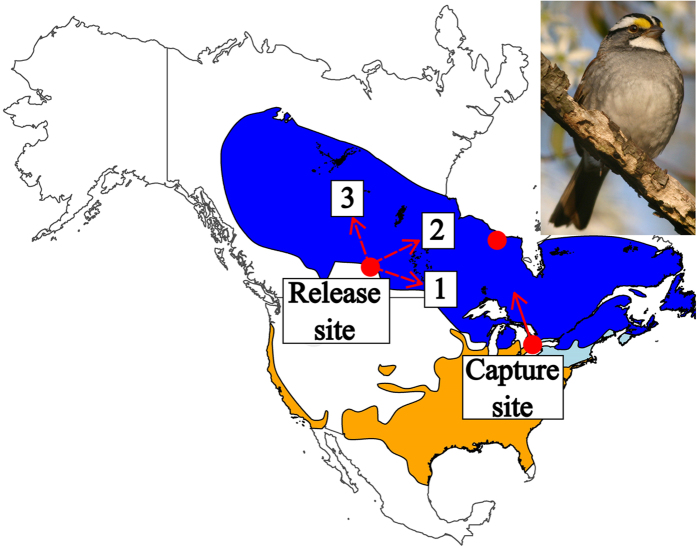
Map of the displacement study showing the distribution of white-throated sparrows^54^ (WTSPs). Colour shading: blue−breeding range, orange−wintering range, light-blue−year-round range. Red dot on Lake Erie (lower right) shows the capture site, Long Point Bird Observatory. The arrow at this dot shows control migratory direction of captured WTSPs inferred by radio-tracking in southern Ontario (Fig. 2b). Red dot in Saskatchewan (upper left) shows location of the displacement and release site. Three dashed line arrows from this dot represent our expectations for the behaviour of the displaced birds as follows: (1) compensation towards the capture site; (2) compensation towards the most northern breeding site inferred by the stable isotope analysis (upper right red dot). Together, scenarios (1) & (2) imply a compensatory response and true navigation performance (flying towards migratory destination from unfamiliar territory^12^). Scenario (3) shows flying parallel to the migratory direction at the capture site and implies no compensation (the lack of true navigation performance). The photo of a WTSP is courtesy of D. Bradley. Map was created using the R^46^ (version 3.2.4, https://www.r-project.org/), package ‘ggmap’.

White-throated sparrows are common ground-foraging, seed-eating migratory songbirds that breed across Canada and overwinter in the U.S., as far south as Florida and as far north as Lake Erie and Lake Michigan^34^ (Fig. 1). They are nocturnal migrants and either travel alone or in sparse flocks^34^,^35^.

From 17−26 April 2014, a total of 69 WTSPs were captured with mist-nets or in ground traps at Long Point Bird Observatory (LPBO), Port Rowan, southern Ontario (42.59 N, 80.40° W). Of the 69 WTSP captured, 31 were identified as second-year (SY), 29 as after-second-year (ASY), and 9 were of unknown age. SY individuals were on their first northward migration, whereas ASY individuals had experienced northward migration at least once before. For most individuals (*n* = 45.65%), we were unable to identify sex based on morphology^36^. Of the 24 birds for which we could identify sex, 21 (87.5%) were male and 3 (12.5%) were female. This sex bias was not surprising given that we captured birds early during spring migration (to allow enough time for surgeries, recovery, and translocation) and males tend to migrate earlier than females^37^. Eight of the 69 individuals captured at LPBO (*n* = 3 SY; *n* = 5 ASY; *n* = 6 males, and *n* = 2 unknown sex) were radio-tagged and released on 26 April to estimate their migratory direction (see details below). The remaining 61 birds were transported on 24 April and 28 April to the Central Animal Facility (CAF) at the University of Guelph, Guelph, Ontario for surgical treatments (see details below).

### Estimating migratory direction at the capture site

We used three independent methods to estimate the migratory direction or destination of birds captured in southern Ontario. The first method, radio-tracking, used different birds from those used for the translocations. The other two methods (stable isotopes and Emlen funnels) sampled the same birds that were later translocated to Saskatchewan.

#### Radio tracking

The birds captured and immediately released with radio transmitters at LPBO were used to estimate the migratory direction of WTSPs passing through the capture site. We attached 1 g NTQB-4-2 digitally coded radio transmitters (LOTEK, Newmarket, ON) using a leg-loop harness^38^. All radio transmitters operated at a frequency of 166.340 MHz and each tag emitted pulses every 1.4, 1.5, or 1.6 sec. We used short inter-pulse intervals to maximize detection probability and we used three different intervals to minimize the probability of signal occlusions (when radio signals from different individuals reach a receiver at the same time). The estimated lifespan of the radio transmitters was 29 d.

**Figure 2:**
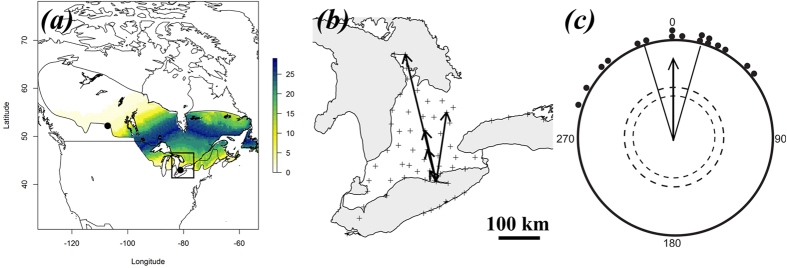
Normal migratory directions of white-throated sparrows (WTSPs) in southern Ontario in spring determined by three methods. (**a**) Map assignment of deuterium isotope ratios extracted from tail feathers grown during the breeding season. Black dots show the capture (lower right) site, Long Point Bird Observatory (LPBO), and displacement (upper left) site in Saskatchewan. The values on the right scale represent the number of birds in the sample that were isotopically consistent with a cell of the same colour in the map representing a likely origin at 2:1 odds (Supplementary Material). Black rectangle shows the spatial extent of the Motus automated-telemetry array (zoomed in on (**b**)). (**b**) Migratory flight tracks (black arrows) detected by radio-tracking in southern Ontario by the Motus automated-telemetry array (black crosses). Note that from 8 birds released at the capture site at LPBO, six were successfully radio-tracked, two of which showed identical flight directions. (**c**) Emlen funnel data obtained from a sub-sample (*n* = 17) of later displaced birds tested at the capture site, LPBO. Black dots at the circle’s perimeter: individual mean directions. The arrow shows mean group vector flanked by its 95% confidence interval (solid lines). The dashed circles indicate the minimum length of the group mean vector needed for significance according to the Rayleigh test^45^ (inner circle, *P* = 0.05; outer, *P* = 0.01). Combined, all three methods indicate that WTSPs migrating through LPBO are heading generally North most probably towards breeding and/or natal sites in central Ontario. Maps were created using the R^46^ (version 3.2.4, https://www.r-project.org/), package ‘ggmap’. The circular diagram was created by Inkscape (version 0.91, https://inkscape.org/).

Movements were tracked using the Motus Wildlife Tracking System (hereafter Motus), a network of automated VHF telemetry towers^39^,^40^ (Fig. 2b; http://motus-wts.org/). At the time of our study, there were 30 telemetry towers throughout southern Ontario (Fig. 2b). Migratory direction for each bird was calculated by drawing a vector connecting LPBO and the location of the VHF tower that last detected the bird.

#### Stable isotope analysis and geographic assignment

To estimate the spring migratory goal of white-throated sparrows, we also analyzed stable-hydrogen isotopes (*δ*^2^H) from the central tail feathers from a sub-sample of randomly chosen WTSPs (*n* = 42). Central tail feathers are grown on or near breeding grounds^36^ and thus contain *δ*^2^H signatures from the breeding grounds the summer prior to capture. Because *δ*^2^H varies predictably over a geographic gradient in North America, we used *δ*^2^H values in tail feathers (*δ*^2^H_f_) to estimate breeding origin using spatially explicit Bayesian assignment techniques^41^. This approach relies on the assumption that birds captured in spring were heading to the same breeding location as the previous year (in the case of ASY birds) or place that they were born (in the case of SY birds). Stable isotope analysis was performed at the National Hydrology Research Centre (Saskatoon, SK). See Supplementary Material for the detailed protocol of the analysis and geographic assignment tests.

#### Orientation tests using Emlen funnels

To estimate migratory directions of WTSPs migrating through LPBO we also performed orientation tests (one test per individual) on a sub-sample (*n* = 26) of the birds that were later translocated. The tests were performed in Emlen funnels^42^ at night and outdoors near the capture site on 23–27 April under clear (95–100% cloud free), moonless skies. Before tests, all birds were placed in outdoors cages for approximately 1 hr at dusk to facilitate compass calibration that is known to occur during sunset^43^,^44^. The mean direction of each bird was estimated from the distribution of the scratches on the inner surface of the funnel. All tests where birds were active and oriented (according to the Rayleigh test of uniformity with uniform distribution as the null hypothesis) were used to calculate a mean group direction^45^. See Supplementary Material for additional details on the Emlen funnel tests.

### Surgical treatments and housing of birds

Birds were randomly assigned to the following 5 groups based on surgical treatments: (1) bilateral sectioning of the ophthalmic branches of trigeminal nerve (*RealMag*), (2) bilateral sectioning of olfactory nerves (*RealOlf*), (3) sham trigeminal ablation (*ShamMag*), (4) sham olfactory ablation (*ShamOlf*), and (5) no treatment (*Intact*). From 44 surgically treated birds, 40 birds successfully survived and recovered, resulting in similar final sample sizes in all four groups (*n* = 11 *RealMag, n* = 9 *RealOlf, n* = 10 *ShamMag, n* = 10 *ShamOlf, n* = 10 *Intact*). See Supplementary Material for detailed surgical protocols.

All birds were held indoors in plastic cages with dimensions of 60 cm × 40 cm × 27 cm at the University of Guelph. The enrichment, shape, size of cages and numbers of individuals in each cage fulfilled Guidelines of the Canadian Council on Animal Care, CCAC Guide to the Care & Use of Experimental Animals and Guidelines to the Use of Wild Birds in Research. Birds were usually kept in pairs to facilitate social interactions and provided with food (mixture of sunflower and millet seeds) and drinking water with added vitamins *ad libitum*.

### Transportation and radio-tracking at displacement site

**Figure 3:**
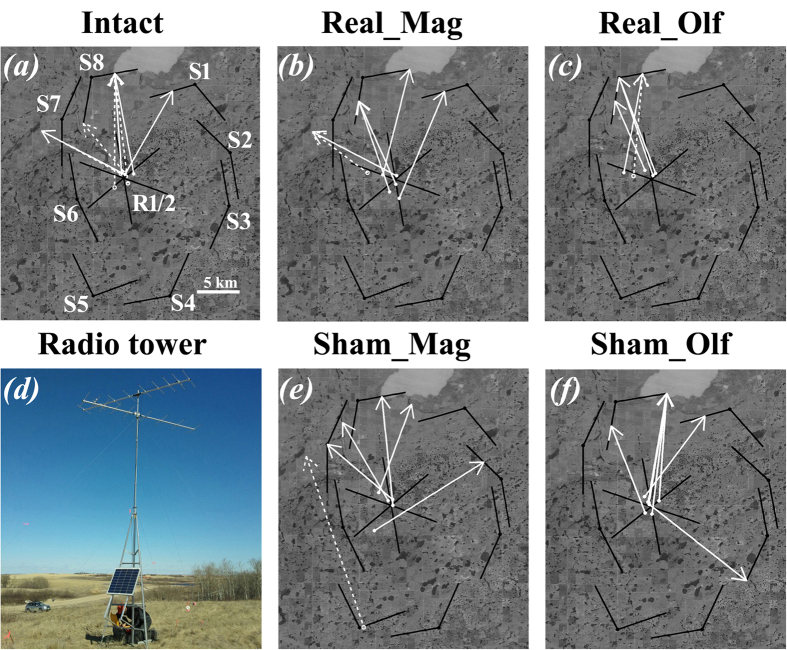
Migratory directions of the birds displaced from southern Ontario and released in central Saskatchewan. (**a**) INTACT−non-surgically treated birds, (**b**) REAL_MAG−birds with ablated ophthalmic branch of trigeminal nerve (the assumed magnetic sense is deactivated), (**c**) REAL_OLF−birds with sectioned olfactory nerve (the sense of smell is deactivated). (**e**) SHAM_OLF and (**f**) SHAM_MAG− birds with sham surgical treatments simulating the surgeries on olfactory and trigeminal nerves, correspondingly, but without real sectioning the nerves. S1–S8 on (**a**) show the eight perimeter VHF towers a photo is shown on (**d**), the courtesy of D. Kishkinev) and R1/2 represents two release site towers. Orientation of the black lines coming from the towers on (**a**–**c**,**e,f**) shows direction where the antennas were pointed to. The length of the black lines shows detection range of each antenna (our conservative assessment as 5 km). Scale bar (the lower right corner) on (**a–c,e,f**) equals 5 km. White arrows on **(a–c,e,f)** show calculated tracks of departing birds drawn as a line connecting the starting location of migratory flight and the last known location detected by VHF towers (solid lines – the starting location was well known, dashed line – the starting location was approximated). Maps were created using the R^46^ (version 3.2.4,  https://www.r-project.org/), package ‘ggmap’.

All birds (*n* = 50; Table S1) were transported in modified pet carriers by air from Guelph, Ontario to Saskatoon, Saskatchewan on 6 May. During transport and prior to release, birds were provided with food and water *ad libitum*. On 9 May, all translocated birds were radio-tagged and subsequently released in a small woodlot, ~45 km northeast of Saskatoon (52.26° N, 106.05° W; Fig. 3).

Radio-transmitters used in Saskatchewan were the same make and model as those used at the capture site (see above). To track translocated birds, we set up an array of ten automated VHF telemetry towers (continuously scanning for radio-tags) (Fig. 3) consisting of two central towers (R1 and R2) near the release site and eight additional perimeter towers positioned in an octagon 7–14 km away from the release site (S1–S8, Fig. 3). The central towers had three 9-element Yagi antennas (pointing in the following directions: 50°, 190° and 290° for R1 tower, and 110°, 230° and 350° for R2 tower) that allowed us to detect local movements and to register vanishing directions during departures from the release site. The perimeter towers were set up at elevated locations covering rolling, open agricultural terrain to maximize detection range and were equipped with two 9-element Yagi antennas oriented in the direction of adjacent neighbouring towers. The detection range of a tower with a 9-element Yagi was conservatively assessed as at least 5 km^40^. Thus, no matter which migratory direction a released bird would take, its vanishing direction would be registered both at the release site and several kilometers after departure (Fig. 3). Data for each tower were downloaded daily.

### Statistical analysis

We used the standard Rayleigh test of uniformity^45^ to assess if a mean group direction significantly differed from a uniform distribution (the null hypothesis). To compare migratory direction among treatment groups, circular statistics were performed using Oriana (version 4.0; http://kovcomp.co.uk/). Differences in mean direction between groups were analysed using the parametric Watson-Williams F-test because the assumptions underlying this test (von Mises distribution, the vector lengths r ≥ 0.75) were fulfilled^45^. To test if the mean group directions tended to cluster around expected goalward-oriented directions (i.e. towards the inferred breeding and/or natal destinations, see Fig. 1), we conducted a V-test^45^. For this test, we compared observed directions from each group to two scenarios of goal-oriented behaviour (Fig. 1): (1) flying from the release site towards the most northern breeding and/or natal site inferred from the stable isotope analysis (Port Severn, Ontario [44.80° N, 79.72° W], Figs 1 and 2a) with an expected direction of 64° (hereafter a great circle or orthodrome direction was used); (2) flying from the displacement site towards the capture site in southern Ontario with an expected direction of 109°. These two scenarios appear to be biologically plausible given a wide range of compensatory responses that have been reported for displaced common cuckoos which showed compensation towards capture sites as well as final and intermediate migratory destinations^11^. To test if the concentration of flight directions was similar between displaced groups, we performed the Equal Kappa test (included in R^46^ package ‘circular’). To test the equality of proportions of displaced birds with different fates (e.g., migratory departure or dead), we performed the Pearson’s chi-square test using R^46^.

## Results

### Orientation and breeding origin of WTSPs captured in southern Ontario

In three independent tests, the inferred migratory direction of birds captured in southern Ontario was generally north (Fig. 2). Of the eight radio-tagged birds, six of (two radio transmitters failed and were excluded from the analysis) released at the capture site (LPBO) were detected by at least one Motus tower^47^, excluding the release site, and showed migratory movements to the north (for detailed tracking data see Table S1). The statistical analysis of departure directions revealed a mean group vector towards a north-northwestern direction (Fig. 2b; α = 344°, *r* = 0.97, *n* = 6, 95% confidence interval [hereafter 95% CI] = 330°–358°; Rayleigh test of uniformity: Z = 5.68, *P* < 0.001).

For the orientation tests using Emlen funnels, two thirds (17/26) of the birds had a mean orientation that differed from the uniform distribution, and their mean group vector was due north (Fig. 2c; α = 360°, *r* = 0.83, *n* = 17, 95% CI = 343°–16°; Rayleigh test of uniformity: Z = 11.78, *P* < 0.001). These results were in agreement with results of the radio-tracking (95% CIs overlapped; Watson-Williams F-test did not show difference between mean group directions: F = 1.19; *P* = 0.29; df1 = 1; df2 = 21).

Using our spatially explicit assignment approach to determine the likely destinations of spring migrating sparrows using *δ*^2^H_f_ values, we identified a band of highest probability of origin as a region of central Ontario coincident with the boreal forest (Fig. 2a). As expected, assignment was generally insensitive to longitude due to the nature of the *δ*^2^H_f_ isotopic contours in eastern North America but was consistent with a fairly constrained band of latitude. The most parsimonious conclusion was that sparrows were then migrating due north from their capture location as shown (Fig. 2b,c).

### Tracking of translocated birds

From 50 birds translocated and released in Saskatchewan, 33 individuals left the detection area of the array within the tracking period of 11 days (10–21 May)^47^. Birds tended to depart in the first half of the night (mean time after sunset = 97 min, s.d. = 50 min). The fates of the remaining birds in which migratory flights were not documented are described in the supplementary material (Table S2).

The mean group directions of *Intact, RealMag, RealOlf*, and *ShamMag* groups were oriented towards the northwest (Fig. 3a–c,e and Table 1), and the mean group direction of the *ShamOlf* group was towards the northeast (Fig. 3f and Table 1). There were no significant differences between mean group directions across all five groups and between any of the displaced groups and the group released at the capture site in Ontario (95% CIs overlapped; for Watson-Williams F-test results see Table S3). In addition, concentrations of individual directions around the mean group direction were not different among all five displaced groups (Equal Kappa Test: χ^2^ = 4.51, *P* = 0.34, df = 4).

The mean group directions of the displaced birds were significantly different from expected goal-ward directions in all cases but one (*ShamOlf* group with the expected direction of 64°, V = 0.51, *P* = 0.03; Table S4). Notably, none of the 95% CIs of the mean group directions in the five groups of displaced birds included the chosen expected directions, which suggests that none of the groups appeared to be goal oriented.

We found no significant difference between the numbers of any surgically-treated birds and the *Intact* group leaving the release site (Pearson’s two-sided chi-squared test: *Intact* vs *RealOlf*: χ^2^ = 0.95, *P* = 0.33; *Intact* vs *RealMag*: χ^2^ = 1.53, *P* = 0.22; *Intact* vs *ShamOlf*: χ^2^ = 0, *P* = 1; *Intact* vs *ShamMag*: χ^2^ = 0.95, *P* = 0.33) or dying for unknown reasons (Pearson’s two-sided chi-squared test: *Intact* vs *RealOlf*: χ^2^ = 0.53, *P* = 0.47; *Intact* vs *RealMag*: χ^2^ = 0.05, *P* = 0.94; *Intact* vs *ShamOlf*: χ^2^ = 0.53, *P* = 0.53; *Intact* vs *ShamMag*: χ^2^ = 0.30, *P* = 0.31), which suggests that surgery had no effect on the birds´ general migratory abilities.

## Discussion

We originally set out to examine two hypotheses to explain the proximate cues migratory songbirds use for navigation. Instead, our results provide evidence that WTSPs during spring migration were unable to display initial re-orientation towards their goal, even when their magnetic and olfactory senses were intact. Regardless of the surgical treatments, all birds that were translocated 2,200 km west-northwest from southern Ontario to Saskatchewan in the spring were either unable to, or chose not to, compensate for displacement and travelled in northerly directions during their initial post-displacement flights. Importantly, this was also the case for birds that were not surgically treated, showing that the surgery itself did not influence orientation behavior. This was unexpected given the number of past studies that have shown compensatory behavior^2^,^7^,^8^,^9^,^10^,^11^ and considering the fact that experienced individuals in a very closely related species, the Gambel’s white-crowned sparrow (*Zonotrichia leucophrys gambelii*), demonstrated a compensatory response after cross-continental displacement during autumn migration^9^.

One explanation for the differences between our results and others^2^,^7^,^8^,^9^,^10^,^11^ could be species-specific migration distance. Gambel’s white-crowned sparrows migrate longer distances (Alaska to Mexico) and, therefore, could be better navigators than WTSPs. However, in a displacement study by Mewaldt^8^, compensatory behavior was also documented in a short-distance migratory subspecies of white-crowned sparrow (*Zonotrichia leucophrys pugetensis*) that migrates from British Columbia to California, as well as in a middle- to short-distance closely related migratory species, the golden-crowned sparrow (*Zonotrichia atricapilla*). It should be noted, however, that Mewaldt^8^ inferred compensatory behavior from individually-marked birds that were recaptured after displacement. Thus, he was unable to track birds that were never recaptured, so it remains unclear whether all, or even a high proportion of, displaced birds showed compensatory behaviour.

It is also possible that our method of detecting initial (first 10–20 km upon release at the displacement site) flight directions was not able to detect a compensatory response if it was manifested later in the migratory flight, beyond the area covered by our telemetry array. Such a difference between initial and later migratory directions could be due to attracting or repelling effect of local geographical landmarks. A late-migration compensatory response was recently reported in displaced lesser black-backed gulls^10^. However, white-crowned sparrows, a close relative of WTSPs, already showed compensatory behaviour after an initial, short-distance (first 5–45 km) flight from their release site, despite the fact that the displaced birds were thousands of kilometers from their wintering grounds^9^. Compensatory orientation responses have also been reported in a few studies where captive songbirds were tested in Emlen funnels after real or magnetically simulated displacements^2^,^21^, suggesting that such responses can be demonstrated immediately after displacement.

Another possibility is that migratory songbirds are only able to show compensatory behavior in autumn when migrating towards their wintering grounds and not during spring when migrating to the breeding grounds^7^,^9^. Although the reason for such seasonal differences is not clear, it could be due to differences in the selective pressures of finding suitable habitat between seasons. Nevertheless, past studies suggest that seasonal differences in compensatory performance are unlikely. Mewaldt^8^ reported that wintering white- and golden-crowned sparrows displaced from California to Louisiana and Maryland compensated for displacement during spring migration and compensatory responses during spring migration have also been reported in Eurasian reed warblers^2^ (*Acrocephalus scirpaceus*).

A fourth possibility is that WTSPs are not motivated to return to their past breeding or natal sites because the benefits of returning to a familiar location are not high. However, past studies on this species suggest they do exhibit some philopatry, especially for males^48^,^49^. Of course, just because a species shows site fidelity does not guarantee that individuals would still be motivated to return to familiar breeding sites after a long-distance displacement, especially when suitable breeding (or non-breeding) habitat is close to the displacement site. However, the birds we displaced likely did not know the location of the closest breeding habitat when they were released (the boreal forest was ~ 50 km directly north). In addition, the migratory orientation and the return rates of translocated white- and golden-crowned sparrows suggests that these closely-related species retain their motivation to fly back to wintering or breeding sites after long-distance displacements (up to 3,900 km^8^,^9^).

The lack of compensatory behavior could be due to social factors we did not directly observe. For example, displaced birds could have been influenced by conspecifics and/or other *Zonotrichia* sparrows that were migrating due north at the time of our experiment^50^. This explanation seems to be unlikely given that WTSPs migrate alone or in loose unstable flocks^35^. Mewaldt^8^ and Thorup *et al*.^9^ used *Zonotrichia* species with similar social behaviour as WTSPs in their displacement experiments and, in all cases, adult birds were able to correct for cross-continental displacements even though they were likely to have encountered conspecifics and/or other related species at release sites. Furthermore, Perdeck^7^ displaced European starlings captured in the Netherlands on autumn migration and released them in Switzerland. Starlings are social birds and readily join conspecifics flocks. However, Perdeck found a difference between navigational strategies of first-time and experienced migrants implying that the former were not relying on conspecifics while making navigational decisions, whereas starlings with migratory experience compensated and were found primarily inside their normal wintering area.

In our view, the most likely explanation for the absence of compensatory behaviour, if indeed birds did not change direction later in migration, is not their general inability to detect a geographic displacement, but rather the natural flexibility of birds’ true navigational behaviour, the manifestation of which could depend on many factors that seem to be largely underappreciated. We suggest that our displaced birds with migratory experience might not use a goalward migratory strategy^7^,^9^,^10^ but instead were following a constant vector or clock-and-compass strategy^9^,^51^, that is they behaved similarly to inexperienced, first-autumn migrants. Daily calculations of current position relative to the goal to perform true navigation seems to be more cognitively demanding than a simple one-direction or vector strategy. Therefore, the constant vector strategy could be adaptive if birds are relatively close to their spring destinations. Birds could also switch from goal-ward true navigation to constant vector strategy if they are habitat generalists on the breeding grounds or have broad east-west stretch of breeding habitat (i.e. the boreal forest). Birds could be motivated to perform navigational calculations only when it is highly profitable, such as when navigational failure leads to a high probability of mortality or when it is critical to find specific habitats. At the last stage of spring migration, WTSPs could rely on a simple ‘fly north until you find familiar landmarks or suitable habitat’ strategy, which could be sufficient for finding suitable breeding habitat, especially for boreal-breeding birds in North America.

The lack of compensatory behavior in migratory songbirds may be more common than previously believed, and one explanation is that ‘negative’ results have been largely overlooked in favour of ‘positive’ results. For example, Perdeck’s early study on European starlings^7^ is widely cited because it provides examples of songbirds demonstrating compensatory behavior (i.e. true navigation). In this study, he displaced adult migrating starlings in autumn from the Netherlands to Switzerland and most recaptures were inside the normal wintering grounds in the Netherlands. However, when adult starlings were displaced a longer distance from the Netherlands to Barcelona, Spain, the results were not nearly as convincing^52^, yet the earlier study is cited far more often than the later study. More recently, two contemporary studies that tracked migratory routes of adult common cuckoos and lesser black-backed gulls after displacement^10^,^11^ showed high variability in compensatory responses^10^. It is possible that new tracking studies will shed more light on how variable navigational performance may be in birds and, as the number of studies grows, hopefully we will gain a better understanding of how life-history and ecological traits influence navigational performance.

In conclusion, our study suggests that displaced migratory birds with migratory experience may not always demonstrate true navigational performance and goalward orientation as is typically expected. We emphasize that our data do not necessarily suggest that WTSPs are unable to perform true navigation but rather that some birds may not to do so at a specific time of the annual cycle. The sensory mechanisms of birds may determine their ability to navigate, i.e. whether they are able or not able to find their position relative to the goal, but many other factors related to the costs and benefits of making compensations after being displaced (e.g., distance to the goal, proximity to suitable habitat), may modify the migratory strategy adopted by an individual. Nevertheless, our results suggest that WTSPs, and perhaps other short-distance migrants with large east-west breeding ranges, may use a constant direction or clock-and-compass strategy when approaching their breeding grounds in spring. If this were the case, naturally-occurring east-west wind drifts and compass errors could facilitate longitudinal colonization and result in large uninterrupted stretches of breeding ranges. It may also explain why short-distance migrants tend to colonize new breeding territories faster compared to long-distance migrants^53^.

## Additional Information

**How to cite this article**: Kishkinev, D. *et al*. Experienced migratory songbirds do not display goal-ward orientation after release following a cross-continental displacement: an automated telemetry study. *Sci. Rep.* **6**, 37326; doi: 10.1038/srep37326 (2016).

**Publisher's note:** Springer Nature remains neutral with regard to jurisdictional claims in published maps and institutional affiliations.

## Supplementary Material

Supplementary Information

## Figures and Tables

**Table 1:** Results of radio-tracking of the displaced experimental white-throated sparrows in Saskatchewan.

Group	Mean group direction (*α*)	Vector length	Sample size (*n*)	Rayleigh uniformity test (Z)	*P*	95% Confidence interval
INTACT	341°	0.87	8	5.99	<0.001	314°–7°
REAL_OLF	348°	0.97	6	5.65	<0.001	333°–3°
REAL_MAG	340°	0.86	6	4.38	<0.01	305°–14°
SHAM_OLF	16°	0.76	7	4.01	0.01	337°–56°
SHAM_MAG	354°	0.82	6	4.08	0.01	316°–32°

INTACT−non-surgically treated birds, REAL_OLF−sectioned olfactory nerve (the sense of smell is deactivated), REAL_MAG−ablated ophthalmic branch of trigeminal nerve (the beak organ’s magnetic sense is deactivated), SHAM_OLF and SHAM_MAG−sham surgical treatments simulating the surgeries on olfactory and trigeminal nerves, correspondingly, but without real sectioning of the nerves.
