# Supplementary material for: Experienced migratory songbirds do not display goal-ward orientation after release following a cross-continental displacement: an automated telemetry study
Source: Sci Rep. 2016 Nov 23;6:37326. doi: 10.1038/srep37326 (PMC5120330; doi:10.1038/srep37326)
Supplement: Supplementary Information [file srep37326-s1.pdf]

Experienced migratory songbirds do not display goal-ward orientation after release following a cross-continental displacement: an automated telemetry study

Dmitry Kishkinev[1,2,*], Dominik Heyers[3], Bradley K. Woodworth[1], Greg W. Mitchell[4], Keith A. Hobson[5,6], and D. Ryan Norris[1]

[1]*Department of Integrative Biology, University of Guelph, 50 Stone Road East, N1G 2W1 Guelph, Ontario, Canada*; [2]*School of Biological Sciences, Bangor University, Deiniol Road, LL57 2UW Bangor, Gwynedd, UK*; [3]*AG Neurosensorik / Animal Navigation, Institute of Biological and Environmental Sciences, University Oldenburg, D-26111 Oldenburg, Germany* [4]*Wildlife Research Division, Environment and Climate Change Canada, National Wildlife Research Centre, 1125 Colonel By Drive, K1H 0H3, Canada, Ottawa, Ontario, Canada*; [5]*Wildlife Research Division, Environment and Climate Change Canada, 11 Innovation Boulevard, S7N 3H5 Saskatoon, Saskatchewan, Canada*; [6]*Department of Biology, University of Western Ontario, N6A 5B7, London, Ontario, Canada*

[*] *Authors for correspondence (dmitry.kishkinev@gmail.com & rnorris@uoguelph.ca)*

Supplementary Material

Stable isotope analysis and geographic assignment

A 2:1 chloroform:methanol solvent rinse was used on the feather probes to remove surface oils. Stable-hydrogen isotope analyses of feathers were conducted using the comparative equilibration method[1] through use of calibrated keratin δ^2H reference materials. Stable-hydrogen isotope measurements were performed on H_2 derived from high-temperature (1350˚C) flash pyrolysis of 350 ± 10 μg feather subsamples using continuous-flow isotope-ratio mass spectrometry. Measurement of two keratin laboratory reference materials (CBS: -197‰, KHS: -54.1‰; corrected for linear instrumental drift) included in each run (n = 5) showed within-run SD values of < 2 ‰. All results are for non-exchangeable δ^2H expressed in the typical delta notation, units per mil (*‰*), and normalized on the Vienna Standard Mean Ocean Water – Standard Light Antarctic Precipitation (VSMOW-SLAP) standard scale.

Following Wunder and Norris[2] and Hobson *et al.*[3], we assigned birds to breeding areas using a likelihood-based assignment method in a spatially explicit framework. We first converted a GIS-based model of expected amount-weighted, growing-season δ^2H in precipitation[4] (hereafter δ^2H_p) into a δ^2H_f model (hereafter isoscape). We employed an algorithm relating variation in δ^2H_f to variation in δ^2H_p as previously described by Hobson *et al.*[5] for ground-foraging, short-distance migrants ($\delta^2H_f = -22.98 + 0.95\ \delta^2H_p$). To limit geographic assignments to biologically plausible areas, we used a digital range map[6] to clip the recalibrated isoscape to the breeding range using functions in the raster package[7] in the R statistical computing environment[8]. For each individual sample, we subsequently assessed the likelihood that each cell in the calibrated isoscape represented a potential origin for the individual using a normal probability density function[2]:

$$f(y^*|\mu_c,\sigma_c)=\left(\frac{1}{\sqrt{2\pi}\sigma_c}\right)\exp\left[-\frac{1}{2\sigma_c^{\ 2}}(y^*-\mu_c)^2\right], \qquad (1)$$

where $f(y^*|\mu_c,\sigma_c)$ represents the probability that a given cell represents a potential origin for an individual for unknown origin (y^*), given the expected mean δ^2H_f for that cell (μ_c) based on the predicted value from the calibrated isoscape, and the expected standard deviation (σ_c) of δ^2H_f of individuals growing their feathers at the same locality. We estimated σ_c using the standard deviation of the residuals from the regression equation reported in Hobson *et al.*[5], i.e., 12.9‰. To estimate probability of origin for a set of N cells, we then normalized the likelihoods calculated using *Eqn 1* as follows:

$$\pi_b=\frac{f(y^*|\mu_c,\sigma_c)}{\sum_{b=1}^{B}f(y^*|\mu_c,\sigma_c)} \qquad (2)$$

This resulted in a set of spatially explicit probability densities for each individual. Based on 2:1 odds that a given assigned bird had truly originated from within that range, we identified the set of cells that defined the upper 67% of estimated 'probabilities of origin' (*eq. 2*) and coded those as 1 and all others as 0. Thus, each bird was simultaneously assigned to multiple potential origins and the results of these assignments were then summed over all individuals.

Emlen funnel tests

Each test lasted for approximately 40 min and started at the end of astronomical twilight (22:10-22:50 local time). We used modified Emlen funnels made of aluminium (top diameter 300 mm, bottom diameter 100 mm, slope 45°) with the top opening covered by netting. The directionality of the birds' activity was recorded as scratches left by their claws on a print film covered with a dried mixture of whitewash and glue. Two researchers (D.K., D.H.) independently estimated each bird's mean direction from the distribution of the scratches. The mean of the two observers' recorded directions was considered the estimate of orientation. If both observers considered the scratches to be randomly distributed or if the two observers assessed mean directions so that they were deviating by more than 30º, the bird was considered not to be oriented in a specific direction. The result of a given test was included only if at least 40 scratches were visible on the print film and a unidirectional mean direction was statistically significant (Rayleigh test of uniformity with uniform distribution as the null hypothesis[9]). Inactive birds (less than 40 scratches) and non-oriented individuals (the mean direction not statistically different from the uniform circular distribution) were excluded from analysis. From the individual directions, a group mean direction was calculated using vector addition[9].

Surgical Protocols

All surgeries were done under full anaesthesia using an intramuscular (i.m.) injection of Medetomidine (Domitor©, 0.1%; 0.1ml/kg body weight) and Ketamine (10%; 0.1ml/kg body weight). Additionally, the general analgesic Meloxicam was administered i.m. (0.5 mg/kg body weight) and local anesthetics (Lidocaine) were used on any incisions to maximize analgesia and to prevent any suffering.

The technique of nerve sectioning was chosen because it represents the only reliable method permanently preventing any sensory input (putative magnetic receptors associated with the trigeminal nerve and the olfactory receptors) to reach the brain. The importance of actually cutting the nerve (instead of using surface anaesthetics for temporary anaesthesia) is supported by Wallraff[10] who demonstrated that only bilateral olfactory nerve section completely eliminates the perception of olfactory stimuli, whereas spraying the nasal cavity with surface anaesthetics used in some magnetoreception studies can lead to highly variable and non-standardized results. The present study was done blind meaning that birds treated by sham and real surgeries were visually not distinguishable, and the experimenters, who did radio-tracking and processed the tracking data, did not know which bird belonged to which group.

Each bird was immobilized and head-fixed in a custom-built holder. For the trigeminal ablation. access to the ophthalmic branch of the trigeminal nerve (V1) was gained through a small incision (8-10 mm) along the dorsal orbital rim following gentle retraction of the eyeball and oculomotor muscles. A ~ 3mm piece of V1 was removed bilaterally. This procedure was identical to the one used previously[11-14] and proved to be effective for preventing re-growth in other songbird species.

For the olfactory ablation, the skin on the bird’s forehead was cut in the median sagittal plane (~10-15 mm). A small window (4-6 mm x 10-12 mm length) was cut into the skull and a piece of pneumatised skull bone between the eyes was temporarily removed so that the olfactory nerves were lying open. ~3mm of both olfactory nerves were removed to prevent re-growth. The piece of skull was returned in its place and the overlying skin sealed with surgical glue (Vetglu©). This procedure has been described previously[11].

To minimize duration of anaesthesia, the effect of Medetomidine was antagonized using Atipamezol (Antisedan©, 0.5%; 0.1ml/kg body weight) immediately after the surgery. Each bird was given at least 72 hrs for recovery before being transported.

Supplementary References

1. Wassenaar, L.I., & Hobson, K.A. Comparative equilibration and online technique for determination of non-exchangeable hydrogen of keratins for use in animal migration studies. *Isotopes in Environmental and Health Studies* **39,** 211–217 (2003).
2. Wunder, M.B., & Norris, D.R. Improved estimates of certainty in stable isotope-based methods for tracking migratory animals. *Ecol. Appl.* **18,** 549–559 (2008). doi: 10.1890/07-0058.1
3. Hobson, K. A., DeMent, S.H., Van Wilgenburg, S. L., & Wassenaar, L. I. Origins of American Kestrels wintering at two southern U.S. sites: an investigation using stable-isotope (δD, δ18O) methods. *J. Rapt. Res.* **43,** 325–337 (2009).
4. Bowen, G. J., Wassenaar, L. I., & Hobson, K. A. Global application of stable hydrogen and oxygen isotopes to wildlife forensics. *Oecologia* **143,** 337−348 (2005). doi:10.1007/s00442-004-1813-y
5. Hobson, K.A., van Wilgenburg, S.L., Wassenaar, L.I., & Larson, K. Linking hydrogen (δ2H) isotopes in feathers and precipitation: sources of variance and consequences for assignment to isoscapes. *PLoS One* **7,** e35137 (2012). doi: 10.1371/journal.pone.0035137
6. Ridgely, R.S., *et al.* Digital distribution maps of the birds of the western hemisphere, version 4.0. BirdLife International and NatureServe (2011) Bird species distribution maps

of the world. (BirdLife International, Cambridge, UK and NatureServe, Arlington, USA, 2011).

7. Hijmans, R.J., & van Etten, J.V. Geographic analysis and modeling with raster data. *R Package version* **2,** 1–25 (2012).

8. R Core Team *R: A Language and Environment for Statistical Computing.* R Foundation for Statistical Computing, Vienna, Austria (2013). ISBN 3-900051-07-0 Available at: http://www.R-project.org/.

9. Batschelet, E. *Circular Statistics in Biology.* (Academic Press, 1981).

10. Wallraff, H.G. Olfactory deprivation in pigeons: examination of methods applied in homing experiments. *Comp. Biochem. Physiol. A* **89,** 621–629 (1988).

11. Gagliardo, A., Ioalè, P., Savini, M., & Wild, J.M. Navigational abilities of adult and experienced homing pigeons deprived of olfactory or trigeminally mediated magnetic information. *J. Exp. Biol.* **212,** 3119–3124 (2009).

12. Zapka, M., *et al.* Visual, but not trigeminal, mediation of magnetic compass information in a migratory bird. *Nature* **461,** 1274–1277 (2009). doi: 10.1038/nature08528

13. Kishkinev, D., Chernetsov, N., Heyers, D., & Mouritsen, H. Migratory reed warblers need intact trigeminal nerve to correct for a 1,000 km eastward displacement. *PLoS One* **8,** e65847 (2013). doi: 10.1371/journal.pone.0065847

14. Lefeldt, N., *et al.* Magnetic field-driven induction of ZENK in the trigeminal system of pigeons (*Columba livia*). *J. R. Soc. Interface.* **11,** 20140777 (2014).

Figure S1. A perimeter automated VHF tower. We used 8 perimeter towers, each of which consisted of a 10 m telescopic mast, 1.5 m tripod, a plastic box with SensoreGnome receiver (see https://sensorgnome.org/), a car battery, a solar panel, coaxial cable leads, aircraft cables, mounting brackets and 2 9-element Yagi antennas pointing towards the two closest perimeter towers. The photo is courtesy of D. Kishkinev.

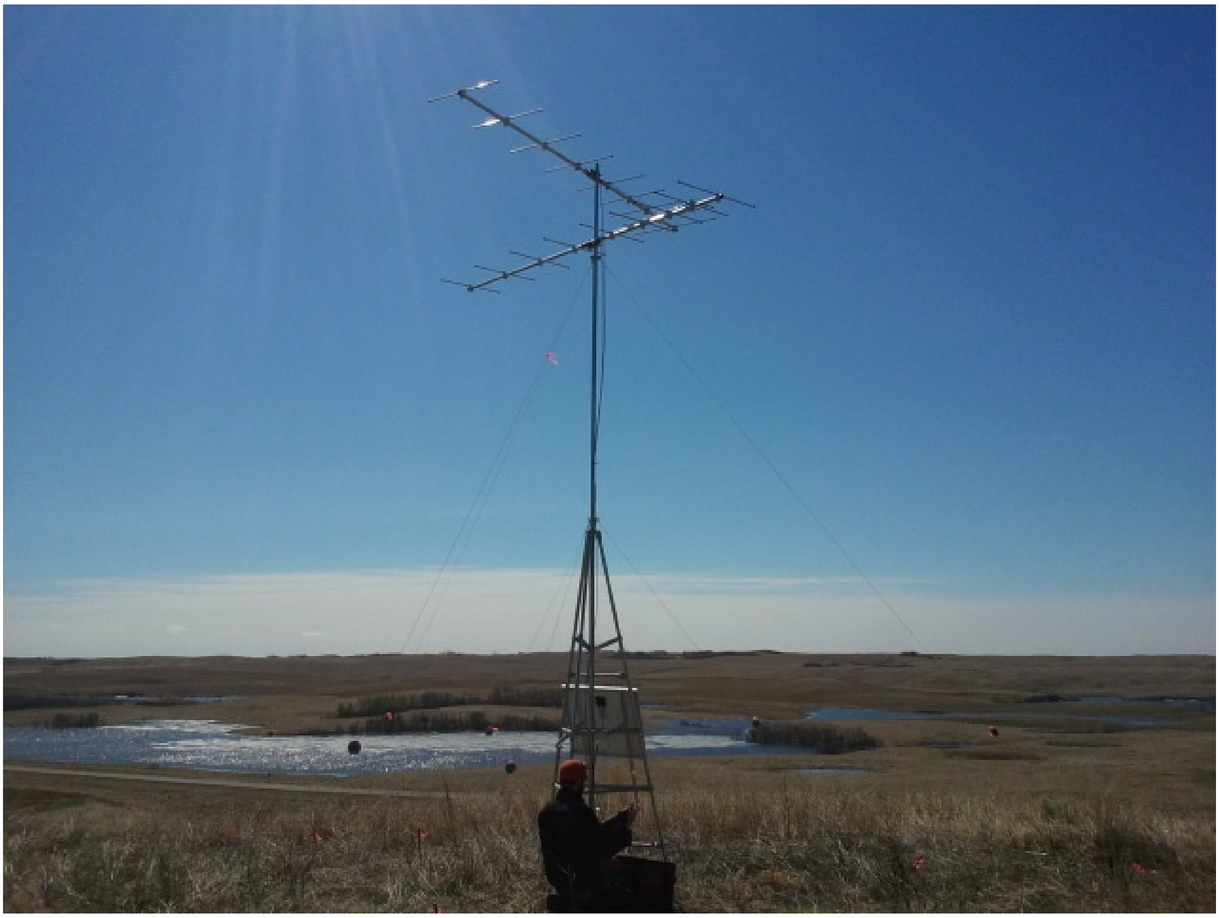

Figure S2. A release site automated VHF tower. There were two release site towers, and each consisted of the same parts as a perimeter tower (*figure S1*) with the only difference that it had 3 9-element Yagi antennas spanning 120°. The azimuth directions the antennas were pointing towards were 60°, 180° and 300° for one release site tower and 360°/0°, 120° and 240° for another release site tower. The photo is courtesy of D. Kishkinev.

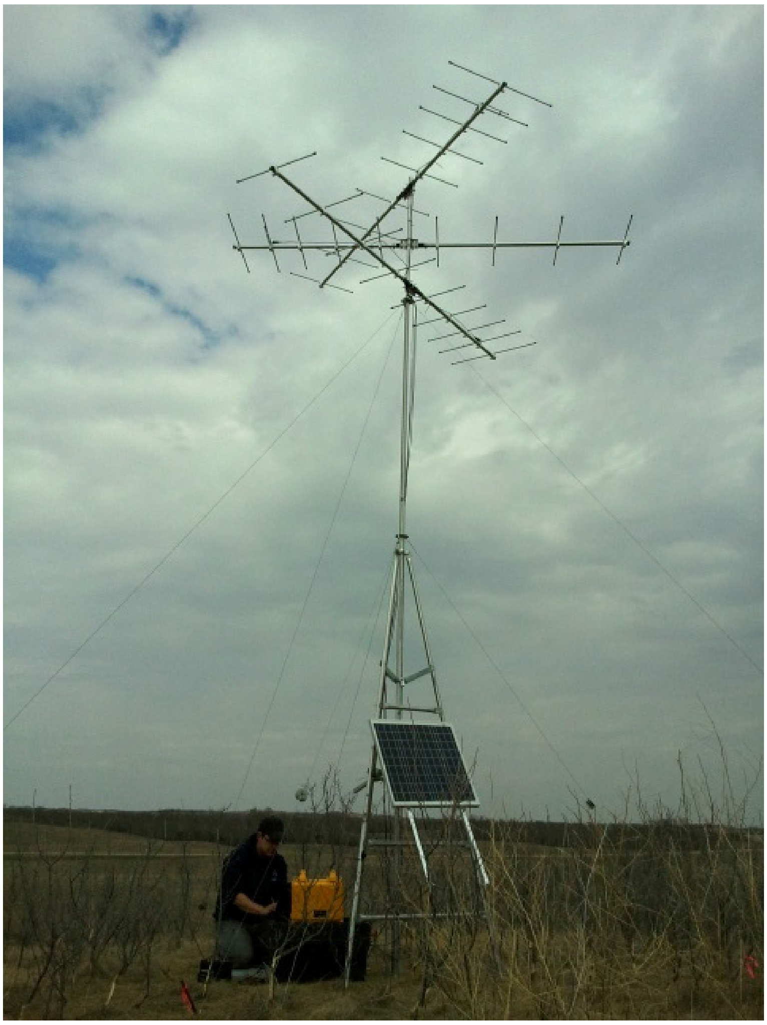

Table S1. Age, sex and calculated migratory directions of the radio-tracked birds. INTACT−non-surgically treated birds, REAL_OLF−sectioned olfactory nerve (the sense of smell is deactivated), REAL_MAG− ablated ophthalmic branch of trigeminal nerve (the beak organ's magnetic sense is deactivated), SHAM_OLF and SHAM_MAG−sham surgical treatments simulating the surgeries on olfactory and trigeminal nerves, correspondingly, but without real sectioning of the nerves. SY−2nd year bird, ASY− after 2nd year, U− unidentified, M−male, F−female.

Group	Ring No	Age	Sex	Migratory direction
INTACT	2541-88 886	ASY	M	349°
	2541-30 399	SY	U	297°
	2541-88 885	U	M	320°
	2541-88 919	ASY	U	298°
	2541-88 920	SY	M	30°
	2541-88 929	SY	M	356°
	2541-88 874	SY	M	353°
	2541-88 884	SY	U	360°
REAL_OLF	2541-88 924	ASY	U	339°
	2541-88 875	SY	U	341°
	2541-88 835	SY	M	337°
	2571-30 384	SY	M	11°
	2581-30 394	ASY	U	5°

	2541-88 854	ASY	U	337°
REAL_MAG	2541-88 882	SY	U	341°
	2541-88 846	ASY	U	336°
	2541-88 864	SY	U	297°
	2571-30 390	ASY	M	23°
	2541-88 819	ASY	M	307°
	2541-88 931	U	F	14°
SHAM_OLF	2541-88 836	ASY	M	340°
	2541-88 867	ASY	F	130°
	2541-88 833	SY	M	4°
	2541-88 895	U	U	9°
	2541-88 890	ASY	U	9°
	2571-30 397	SY	U	7°
	2541-88 932	SY	F	35°
SHAM_MAG	2541-88 881	SY	U	310°
	2541-88 871	U	M	330°
	2541-88 925	ASY	U	341°
	2541-88 820	SY	U	58°
	2541-88 889	U	U	20°
	2541-88 826	SY	U	354°
ONTARIO	2541-88 989	ASY	U	344°
	2541-88 988	SY	M	346°
	2541-88 994	ASY	M	329°

	2541-88 997	SY	M	329°
	2541-89 000	ASY	U	347°
	2571-30 402	ASY	M	9°
	2571-30 406	SY	M	347°

Table S2. End points of the displaced radio-tracked birds in Saskatchewan. INTACT−non-surgically treated birds, REAL_OLF−sectioned olfactory nerve (the sense of smell is deactivated), REAL_MAG− ablated ophthalmic branch of trigeminal nerve (the beak organ's magnetic sense is deactivated), SHAM_OLF and SHAM_MAG−sham surgical treatments simulating the surgeries on olfactory and trigeminal nerves, correspondingly, but without real sectioning the nerves.

Group	Released	Departed for migration	Stayed near release site	Found dead	Unknown fate
INTACT	10	8	0	1	1
REAL_OLF	9	6	0	2	1
REAL_MAG	11	6	0	1	4
SHAM_OLF	10	8	0	2	0
SHAM_MAG	10	6	1	0	3
TOTAL	50	34	1	6	9

Table S3. Results of the Watson-Williams F-test comparisons for mean group directions of the displaced birds. INTACT–non-surgically treated birds, REAL_OLF–sectioned olfactory nerve (the sense of smell is deactivated), REAL_MAG– ablated ophthalmic branch of trigeminal nerve (the beak organ's magnetic sense is deactivated), SHAM_OLF and SHAM_MAG–sham surgical treatments simulating the surgeries on olfactory and trigeminal nerves, correspondingly, but without real sectioning the nerves.

Variables (and N of observations)	F value	*P*	df	df2	Est. Mean
INTACT & REAL_OLF (8 & 6)	0.29	0.60	1	12	344
INTACT & REAL_MAG (8 & 6)	0.00	0.96	1	12	340
INTACT & SHAM_OLF (8 & 7)	3.14	0.10	1	13	356
INTACT & SHAM_MAG (8 & 6)	0.51	0.49	1	12	346
REAL_OLF & REAL_MAG (6 & 6)	0.32	0.58	1	10	344
REAL_OLF & SHAM_OLF (6 & 7)	2.21	0.17	1	11	2
REAL_OLF & SHAM_MAG (6 & 6)	0.13	0.73	1	10	351

REAL_MAG & SHAM_OLF (6 & 7)	2.67	0.13	1	11	358
REAL_MAG & SHAM_MAG (6 & 6)	0.48	0.50	1	10	347
SHAM_OLF & SHAM_MAG (7 & 6)	0.91	0.36	1	11	6
ONTARIO & INTACT (7 & 8)	0.08	0.78	1	13	342
ONTARIO & REAL_OLF (7 & 6)	0.23	0.64	1	11	346
ONTARIO & REAL_MAG (7 & 6)	0.12	0.74	1	11	342
ONTARIO & SHAM_OLF (7 & 7)	3.34	0.09	1	12	358
ONTARIO & SHAM_MAG (7 & 6)	0.41	0.54	1	11	348

Table S4. Results of V-tests testing if mean group directions were goal-ward, i.e. leading towards breeding and/or natal sites into two expected directions based on the following scenarios: (1) 64°−flying from the release site towards the most northern breeding and/or natal site inferred from the stable isotope analysis (Port Severn, Ontario, [44.80° N, 79.72° W], *figure 1, 2a*, hereafter a great circle or orthodrome direction was used); (2) 109°−flying from the displacement site in Saskatchewan towards the capture site. INTACT−non-surgically treated birds, REAL_OLF−sectioned olfactory nerve (the sense of smell is deactivated), REAL_MAG−ablated ophthalmic branch of trigeminal nerve (the beak organ's magnetic sense is deactivated), SHAM_OLF and SHAM_MAG−sham surgical treatments simulating the surgeries on olfactory and trigeminal nerves, correspondingly, but without real sectioning the nerves.

Group	Expected direction	V	*P*
INTACT	64°	0.10	0.35
	109°	-0.54	0.99
REAL_OLF	64°	0.24	0.21
	109°	0.50	0.96
REAL_MAG	64°	0.08	0.39
	109°	-0.54	0.97
SHAM_OLF	64°	0.51	0.03
	109°	0.51	0.03
SHAM_MAG	64°	0.28	0.17
	109°	-0.35	0.88
